# Epidemiology, morbidity and mortality from fall-related injuries in the United Arab Emirates

**DOI:** 10.1186/s13049-014-0051-5

**Published:** 2014-09-02

**Authors:** Michal Grivna, Hani O Eid, Fikri M Abu-Zidan

**Affiliations:** 1 Institute of Public Health, College of Medicine and Health Sciences, UAE University, Al Ain, United Arab Emirates; 2 Trauma Group, Department of Surgery, College of Medicine and Health Sciences, UAE University, Al Ain, United Arab Emirates

**Keywords:** Falls, Elderly, Injury, Prevention, United Arab Emirates

## Abstract

**Background:**

Unintentional falls are a major cause of morbidity and mortality with a significant burden on victims, families, and societies. We aimed to study the mechanism, risk factors, and outcome of hospitalized patients with fall-related injuries in order to propose preventive measures.

**Methods:**

Fall-related injured patients who were admitted to Al Ain Hospital, United Arab Emirates (UAE) for more than 24 hours or who died after arrival to the hospital, were studied over 3 years. Demography, location and time of injury, affected body regions, hospital and ICU stay, and outcome were analyzed.

**Results:**

882 patients were studied, 82% were males, and 22% were less than 19 years old. Majority were from the Indian subcontinent. The most common location for fall injuries was work. Patients injured at work were older and mainly non-UAE nationals (p < 0.0001) when compared with those injured at home. Patients falling from height, when compared with those falling from same level, were older (p = 0.017), had more males (p < 0.001), were mainly from the Indian subcontinent (p < 0.001), had higher ISS (p = 0.011) and longer total hospital stay (p < 0.001).

**Conclusions:**

Falls are a major health problem in the UAE. Falls at work can be prevented by safety education tailored to different ethnic groups, and proper legislation and regulation. Environmental modification using evidence-based architectural design may prevent falls among vulnerable risk groups.

## Background

Unintentional falls are a major cause of morbidity and mortality with a significant burden on victims, families, and societies [1]. They are the second leading cause of injury-related hospitalization for all ages, accounting for about 30-40% of injury admissions [2],[3]. Falls present 10-15% of all Emergency Department visits [4]. More than 60% of injury-related hospitalizations of the elderly is due to falls which cause hip fractures, traumatic brain injuries and upper limb injuries [2],[3]. The cost of fall-related injuries and their effects on the health care systems are high [5],[6].

United Arab Emirates (UAE) is a fast developing country with a population of more than 6 million. Falls were the second cause of trauma deaths following road traffic collisions with a death incidence rate of 7.4 per 100 000 population [7]. The personal, environmental, and equipment risk factors for falls vary in different communities. It is important to conduct proper epidemiological studies if we aim to propose useful preventive measures. There is little information on falls in the Gulf area of the Middle East. We aimed to study the mechanism of injury, severity and outcome of hospitalized fall-related injuries in the UAE in order to give recommendations regarding their prevention.

## Methods

### Ethics statement

The Local Ethics Committee of Al Ain Health District Area has approved data collection for all trauma patients who were admitted to Al Ain Hospital or who died in the Emergency Department (UAE RECA/02/44). Data were collected prospectively on a specially designed hard copy form. All patients or their care givers signed a consent form for permitting the use of anonymous data for research or audit.

We studied all fall-related injured patients who were admitted to Al Ain Hospital for more than 24 hours, or who died after arrival to the hospital during the period of March 2003 – March 2006. Al Ain hospital is a major hospital of Al Ain City, which has a population of about half a million [8]. Data were retrieved from Al Ain Hospital Trauma Registry, which were prospectively collected by a full time Research Fellow. Variables studied included age, gender, nationality, location of fall, type of fall (from height or same level), time and date of the fall, injured anatomical body regions, intensive care unit (ICU) admission, duration of hospital stay, and mortality.

Severity of injury of the affected body region was assessed by the Abbreviated Injury Scale (AIS) and by the Injury Severity Score (ISS) [9],[10]. The ISS was calculated manually using the Abbreviated Injury Scale Handbook [11]. The AIS has a value of 1–5 while ISS has a value of 1–75.

### Statistical analysis

Data were analyzed using the Statistical Package for the Social Sciences (IBM-SPSS version 20, Chicago, Il). The Mann–Whitney U-test or Fisher’s exact test were used to compare two independent groups of continuous, ordinal, or categorical data. The Kruskal-Wallis non parametric test was used to compare continuous or ordinal data for more than two independent groups. Probabilities of less than 0.05 were accepted as statistically significant.

We estimated the incidence rates using 2005 UAE census data with an assumption that Al Ain City population structure is similar to that of the whole UAE [8].

## Results

**Figure 1:**
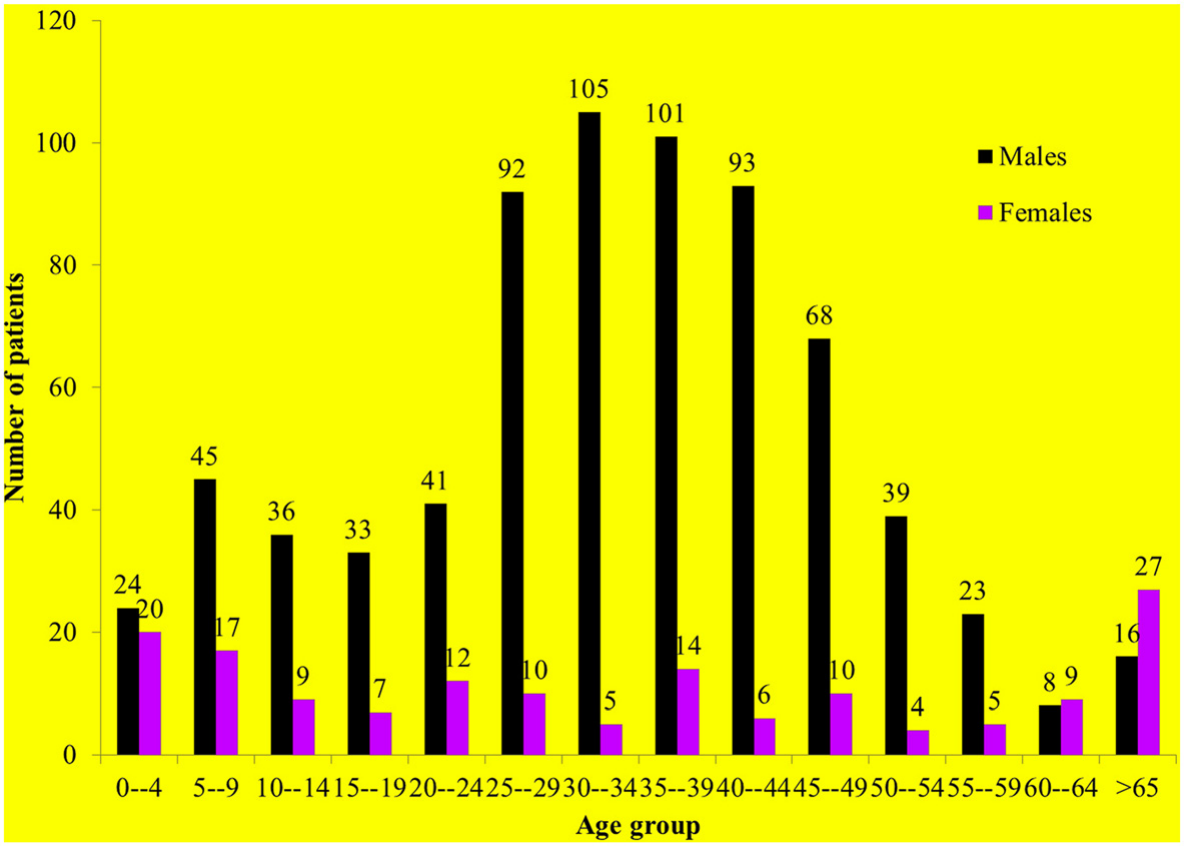
Age distribution of fall patients by gender (n = 727 males, n = 155 females), males = black bars; females = purple bars.

There were 882 patients, 727 males (82%) (male:female ratio was 4.7:1). Mean (SD) age was 32.8 (17.4) years, majority were adults in the productive years of 20–54 years old (68%; n = 600). Twenty two percent (n = 187) were children and youth <19 years (Figure 1). Majority of patients were from the Indian subcontinent (52%; n = 454), followed by Arabs (25%; n = 217), UAE nationals (15%; n = 129), other Asians (5%; n = 47), and others 3% (n = 27).

Work was the most common location for fall injury (51%; n = 451), followed by home (37%; n = 324) and others (12%; n = 107). Patients injured at work were significantly older than those injured at home or other locations (p < 0.0001) (Table 1). Females and UAE nationals were significantly more injured at home compared with males and non-UAE nationals (p < 0.0001) (Table 1). Other locations included road, off-road, public buildings, public area, hotels/clubs and others (12%; n = 107).

**Table 1:** Location of falls by age, gender and nationality, Al Ain Hospital, 2003–2006 (n = 882)

**Variable**		**Home n = 324**	**Work n = 451**	**Other n = 107**	p-value*
Age		26 (1–100)	35 (4–76)	20.0 (3–75)	<0.0001
Gender	Male	186 (57.4%)	448 (99.3%)	93 (86.9%)	<0.0001
	Female	138 (42.6%)	3 (0.7%)	14 (13.1%)	
Nationality	UAE	94 (29.3%)	7 (1.6%)	28 (26.4%)	<0.0001
	Non-UAE	227 (70.7%)	440 (98.4%)	78 (73.6%)	

*p = Kruskall Wallis test or Fisher’s Exact test as appropriate.

Data are presented as median (range) and number (%) as appropriate.

Data on nationality were missing in three patients falling at home, 4 patients falling at work, and one patient falling elsewhere.

There were more patients with falls from height (57%; n = 501) than from same level (43%; n = 381) (Table 2). Patients falling from height, when compared with those falling from same level, were older (p = 0.017), had more males (p < 0.001), were mainly from the Indian subcontinent (p < 0.001), had higher ISS (p = 0.011), were more admitted to the ICU (p = 0.001), and had longer total hospital stay (p < 0.001) (Table 2). Falls from the same level occured more at home (n = 214/324; 66%), while falls from height were more common at work (n = 360/451; 80%). Two patients were injured intentionally during a conflict.

**Table 2:** Demographic and severity variables by type of falls, Al Ain Hospital, 2003–2006 (n = 882)

**Variable**	**Fall from same level (n = 381)**	**Fall from height (n = 501)**	p-value*
Age (years)	31 (1–95)	34 (2–100)	0.017
Gender (male)	273 (71.7%)	454 (90.6%)	<0.001
Nationality (Indian subcontinent)	141 (37.1%)^a^	313 (63.4%)^a^	<0.001
ICU admission	4 (1%)	26 (5.2%)	0.001
Length of hospital stay (days)	5 (1–85)	6 (1–150)	<0.001
ISS	4 (1–25)	4 (1–34)	0.011

*p = Fisher’s Exact test or Mann Whitney test as appropriate; Data are presented as number (%) or median (range) as appropriate.

a = NB. Data on nationality were missing in one patient falling from same level and seven patients falling from height.

**Figure 2:**
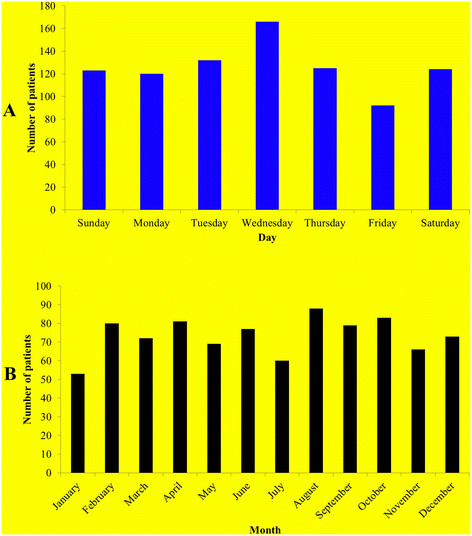
Distribution of fall patients by day of the week (blue bars, A) and month of the year (black bars, B).

**Figure 3:**
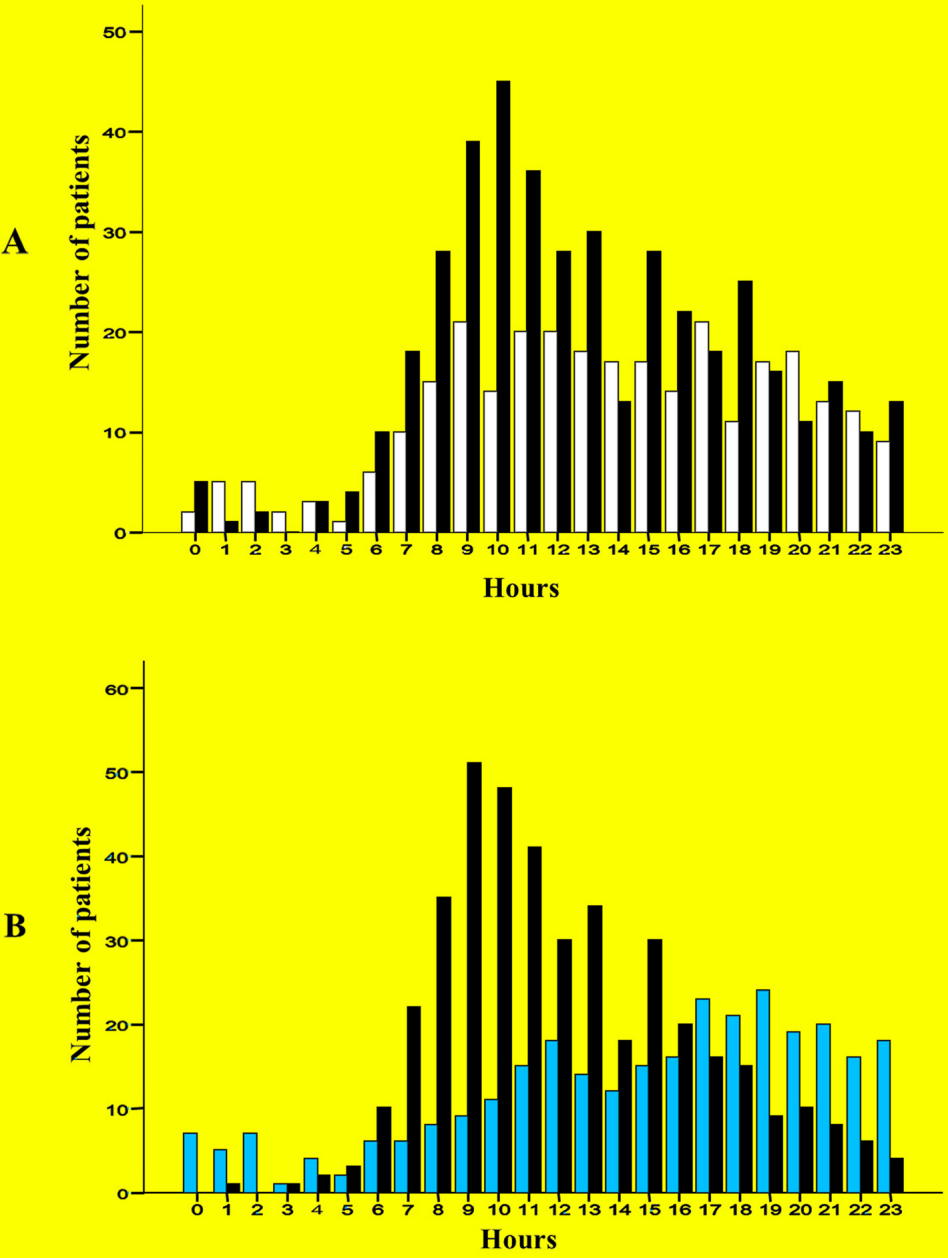
Time distribution of fall patients by mechanism (A) (n = 420 fall from height, n = 291 fall from same level), fall from height = black bars, fall from same level = white bars; and by location of trauma (B) (n = 414 work, n = 297 home), work = black bars, home = light blue bars.

Wednesday had the highest incidence of falls compared with other days (19%; n = 166) (Figure 2A). Twenty six percent (n = 228) of falls occured during the fall (September -November) (Figure 2B). Figure 3 shows the time distribution of falls by mechanism (A) and location (B) of injury. It is clear that fall from height had the highest peak at 9–11 am and occured mainly at work, while fall at the same level occured continuously during daily activity time (8 am – 10 pm) at home. The estimated annual incidence of hospitalized injuries with falls in Al Ain city was 64.3/100,000 persons per year.

The most common injured anatomical region was the lower extremity (n = 362/882; 41%), followed by the upper extremity (n = 260/882; 29%), head/face and neck (n = 157/882; 18%), spine (n = 115/882; 13%), chest (n = 113/882; 13%) and abdomen (n = 34/882; 4%). Patients who fell from height had significantly more injured chest (p < 0.001) and spine (p < 0.001) compared with those who fell on the same level (Table 3). There was a trend for more severe head injury for those who fell from height (p = 0.09). Those who fell on the same level had more severe lower limb injuries compared with those who fell from height (p < 0.04).

**Table 3:** Comparison of location and severity of anatomical regions between fall from the same level and fall from height of hospitalized fall-related injured patients, Al Ain Hospital, 2003–2006 (n = 1041 regions in 882 patients)

**Injury severity by maximum AIS**										
	**Same level (n = 381)**	**From height (n = 501)**		**Same level (n = 381)**			**From height (n = 501)**			
**Region**	**Number (%)**	**Number (%)**	p value*	**Mean**	**Median**	**Range**	**Mean**	**Median**	**Range**	p value*
Head/face/neck	63 (16.5)	94 (18.8)	0.44	1.51	1	1-4	1.83	1	1-4	0.09
Chest	32 (8.4)	81 (16.2)	<0.001	1.66	2	1-3	1.69	1	1-4	0.86
Abdomen	13 (3.4)	21 (4.2)	0.68	1.38	1	1-3	1.81	2	1-3	0.11
Spine	25 (6.6)	90 (18.0)	<0.001	2	2	2-2	2.17	2	2-5	0.36
Upper extremity	117 (30.7)	143 (28.5)	0.53	1.92	2	1-3	1.85	2	1-3	0.18
Lower extremity	151 (39.6)	211 (42.1)	0.50	2.26	2	1-3	2.16	2	1-3	0.04

*p = Fisher’s Exact test or Mann Whitney test as appropriate; Data are presented as number (%) or median (range) as appropriate.

The mean (SD) total hospital stay was 8.6 (11.2) days. Two patients died (0.2%), one falling from a height and another from the same level.

## Discussion

Our study has shown that the highest risk for falls for UAE nationals and females was at home, while work-related falls were more for expatriate males. Falls from the same level occured more often at home while falls from height were more common at work. Patients falling from height had more severe head injuries, were more admitted to the ICU, and had longer hospital stay when compared with those falling from the same level.

Similar to others, majority of our injured patients were males from the Indian subcontinent who were injured at work [5],[12]. Information about occupation was missing in our study. In comparison, construction industry was regarded as the most hazardous industry in Kuwait [13]. Immigrant workers at construction sites are at high risk of injury [5],[14],[15]. They often lack safety equipment and safety education using their own language. We have previously reported the challenges faced with promoting occupational safety in the UAE [7],[16].

Two daily time peaks for falls were identified. They were 9–11 a.m. at work and 5–9 p.m. at home. A study from Canada reported that 11 a.m. was the peak for occupational injuries possibly due to sleep deprivation [17]. Work at the construction sites in the UAE starts earlier because of the hot climate with possible increase in tiredness and sleepiness. The mid-day break for summer months (12.30-4 pm) was introduced in 2005 so as to avoid heat-related illness. This implies that work finishes late with possible reduction in sleeping hours. Dinges et al. have shown that excessive sleepiness reduces performance and ability to stay alert, and increases the risk of errors [18]. The increased home falls at evening is possibly related to activities like dinner preparation. Falls occured more often on Wednesday which was the last working day of the week in the UAE during the study period. Thursday became the last working day of the week in the UAE on 1st September 2006 [19]. We have noticed the same time of increased injury in pedestrian and bicycle-related injuries [20],[21]. The monthly incidence of falls in our study was high during August, similar to a study from Canada [17]. High outdoor temperatures, which can reach up to 50°C, can decrease the vigilance among workers.

Fall from height occured more at work. Falling from height at construction sites was common in Qatar with significant effects on the health care system [5]. A similar study from Taiwan showed that falls were the most common cause of death at workplace [22]. A study from UK reported that slipping and tripping was the most common injury at work [23].

Falls are the leading cause of pediatric injury-related hospitalization [24],[25]. Twenty two percent of patients in our study were children or youth. Families in the UAE tend to have more children who are often supervised by maids or older siblings without enough experience on first aid or safety precautions. Cases of fatal falls from higher level windows and balconies in the UAE highlighted the importance of automatic protection by passive measures, e.g. mandatory instalations of window guards [26]. A study from USA, on pediatric falls from apartment balconies and windows, found that balcony railings with more than 10 cm apart, and windows that were within 60 cm of the floor were the two most important hazards [27]. Children were previously used as camel jockeys in the UAE. They used to have major head injuries [28]. Succesful legislative intervention with substitution of child riders by robots eliminated these injuries [29].

Falls from the same level in our study occured more at home. Falls among the elderly, including same-level falls, can cause serious injuries and death [30]. Five percent of our patients were of more than 65 years old. 30% to 60% of old people fall every year and half of them have multiple falls [31],[32]. Sterling et al. reported that injury severity and death for same-level falls in the elderly was twice that of the young people [30]. Home hazards for falls in the elderly include narrow steps, stairs with four or more steps, absence of railings on stairs, slippery surfaces, and insufficient lighting [33],[34]. Physical environment risks are related to falls [35]. A European study showed that missing window guards at the second level or higher was a major hazard to death and disability [36].

The most common injured region in our study was the extremity, compared with the head in Qatar [5]. Head/face/neck in our study was the second common injured region (18%) with more severe head injury for those falling from height. This indicates the need for safety measures, such as using helmets and installing safety barriers. The annual incidence of fall-related injuries (64.3/100,000) in our study was lower than in Qatar (86.7/100,000), despite that they included only occupational-related falls [5].

It is important to highlight that there are certain limitations in our study. Mortality was low (0.2%). It is possible that patients with more severe injuries may have died before arriving to the hospital. In contrast, patients who had minor fall injuries were treated at the Emergency Department and discharged home. Furthermore, our Trauma Registry was a time-limited research project, which may not reflect the current situation and may be not generalizable to all UAE. Our study was based in Al Ain City, which does not have many high-rise buildings. In contrast Dubai and Abu Dhabi cities have very high buildings with more construction sites and younger labourers, so, the prevalence of falling from height may be different. Furthermore, our registry lacks data on the activity of the person during the fall, type of occupation, the detailed location of the fall at home, socio-economic variables and information on risk-taking behaviors.

## Conclusions

Young males are at high risk for falls at work. Falls at work can be prevented by occupational safety education, proper legislation and regulations. Safety education in the UAE should be tailored to different ethnic groups residing and working in the UAE. Environmental modification using evidence-based architectural design can prevent home falls among vulnerable risk groups such as children and elderly.

## Competing interests

All authors declare that they have no competing interests.

## Authors’ contributions

Conceived and designed the experiments: MG HOE FAZ. Performed the experiments: HOE. Analyzed the data: MG HOE FAZ. Contributed reagents/materials/analysis tools: MG HOE FAZ. Wrote the paper: MG FAZ. Critically read the paper: MG HOE FAZ. Approved final version: MG HOE FAZ. All authors read and approved the final manuscript.
